# Leveraging Machine Learning to Understand How Emotions Influence Equity Related Education: Quasi-Experimental Study

**DOI:** 10.2196/33934

**Published:** 2022-03-30

**Authors:** Javeed Sukhera, Hasan Ahmed

**Affiliations:** 1 Institute of Living Hartford Hospital Hartford, CT United States; 2 Centre for Education Research and Innovation, Western University London, ON Canada

**Keywords:** bias, equity, sentiment analysis, medical education, emotion, education

## Abstract

**Background:**

Teaching and learning about topics such as bias are challenging due to the emotional nature of bias-related discourse. However, emotions can be challenging to study in health professions education for numerous reasons. With the emergence of machine learning and natural language processing, sentiment analysis (SA) has the potential to bridge the gap.

**Objective:**

To improve our understanding of the role of emotions in bias-related discourse, we developed and conducted a SA of bias-related discourse among health professionals.

**Methods:**

We conducted a 2-stage quasi-experimental study. First, we developed a SA (algorithm) within an existing archive of interviews with health professionals about bias. SA refers to a mechanism of analysis that evaluates the sentiment of textual data by assigning scores to textual components and calculating and assigning a sentiment value to the text. Next, we applied our SA algorithm to an archive of social media discourse on Twitter that contained equity-related hashtags to compare sentiment among health professionals and the general population.

**Results:**

When tested on the initial archive, our SA algorithm was highly accurate compared to human scoring of sentiment. An analysis of bias-related social media discourse demonstrated that health professional tweets (n=555) were less neutral than the general population (n=6680) when discussing social issues on professionally associated accounts (*χ*^2^ [2, n=555)]=35.455; *P*<.001), suggesting that health professionals attach more sentiment to their posts on Twitter than seen in the general population.

**Conclusions:**

The finding that health professionals are more likely to show and convey emotions regarding equity-related issues on social media has implications for teaching and learning about sensitive topics related to health professions education. Such emotions must therefore be considered in the design, delivery, and evaluation of equity and bias-related education.

## Introduction

Research on addressing bias in health professionals found that feedback conversations about topics such as bias provoked defensive reactions [1,2]. However, these emotions did not hijack the learning process as learners still perceived their experience as positive while perceiving feedback about their biases as actionable [3]. This finding was unique in the feedback literature, which generally suggests that feedback should be targeted away from the self to avoid hijacking the feedback process [4]. This paradox suggests the need to further explore how emotions may mediate conversations about bias among health professionals.

Understanding the role of emotions when discussing topics related to bias or equity is essential to advance education in the field. We know that emotions play an important role in mediating the relationship between self-concept and learning. If confronted with their biases, learners may perceive a threat and therefore perceive the situation to have a negative attainment value leading to negative emotions. Negative emotions may then impede information recall and promote avoidance in processing its content [5]. Not all emotions have a negative influence on learning. For example, emotions are essential for transformative learning and similar methods that require dissonance, critical reflection, facilitated dialogue, action, and behavior change [6].

The importance of understanding emotions related to bias or equity education is especially salient when defensive or skeptical reactions are provoked. When challenging learners’ perceptions regarding the erroneous beliefs that they are not biased, emotions can lead to the backfire effect, strengthening the belief in such erroneous information even after attempted refutation [7,8]. This could lead learners to expend considerable cognitive resources to counter refutation [9,10] and activate more evidence that supports their original erroneous beliefs.

In our previous work, we found that the idea of having bias and therefore being vulnerable to its effects was a threat to the strongly held belief among health professionals that they must operate without bias [11]. Research suggests that strongly held beliefs, such as the idea that health professionals cannot have bias, are integral to health professionals’ sense of self [12,13]. Bias acceptance, therefore, may be perceived as identity threatening and trigger self-protective responses such as defensiveness and denial [14] to restore a sense of self-worth [15].

Research regarding emotions in health professions education can also be challenging for numerous reasons. For example, there are tensions in how emotions are conceptualized in health professions education. Some view emotions as a physiological response, others as skills or abilities, and others view emotions as a sociocultural mediator [16]. There are also ontological tensions and a lack of conceptual and methodological consistency [17]. Despite such challenges, a deeper understanding of how emotions influence learning is needed to enhance teaching and learning about emotionally challenging topics such as equity.

Advances in machine learning (ML) technology such as natural language processing (NLP) and sentiment analysis (SA) may provide a novel way of approaching such research [18]. ML techniques can automate information processing and have been applied towards applications such as competence assessments [19]. NLP is a form of ML that can structure and extract text-based information making it available for further analysis [20]. NLP and advanced text analytics are being used increasingly in a health care context [21,22]. SA is a mechanism of analysis that evaluates the sentiment of textual data by assigning scores to textual components and calculating and assigning a sentiment value to the text [23].

SA is most commonly discussed in business settings as it allows one to determine customers' overall sentiment about products and services through data scraping and analysis from social media [24]. In health care, SA has been used to analyze online comments regarding hospital services to explore patient experiences [25] and applied to electronic health records to analyze health professional behavior [26]. In another study, SA was applied to twitter health news to compare whether health news is delivered in a manner more consistent with facts or opinion [27]. In these examples, researchers acknowledged their lack of clinical experience and limitations in the execution of their analysis. For example, Gohil and colleagues acknowledge that their methods had not been tested for accuracy [26]. The potential for SA in health professions education research is therefore limited without further research and evaluation.

Our previous research on emotions and bias-related feedback may provide a window into the application of SA. More recently, a shift from in-class to online discussions on sensitive and emotionally charged topics may provide an opportunity for inquiry. A deeper analysis of the language used by health professionals on social media may therefore provide insight into the emotions associated with teaching and learning about equity and bias.

Overall, our aim for this study was to improve our understanding of the role of emotions in bias-related discourse. We, therefore, conducted an SA of bias-related dialogue among health professionals. First, we tested if our SA algorithm was accurate by testing the accuracy of our NLP library on an existing archive of bias-related discourse among health professionals. Second, we utilized our SA to compare if the sentiment toward equity-related online discourse differed between health professionals and the general population.

## Methods

### Sentiment Analysis

Sentiment is a thought, opinion, or idea based on the underlying feeling or emotion about a specific topic or item. SA is utilized to analyze text and assign the writer’s attitude as positive, negative, or neutral given the presence of certain keywords. First, the text is split into four basic components: tokens, sentences, phrases, and entities. Next, an algorithm is applied using one of two systems. In a rule-based system, rules are manually crafted to analyze textual components. Specific words are scored as negative, neutral, or positive and associated with a score. These values are then tabulated to provide an estimate of the overall sentiment of the text. In an automatic system, machine learning technology is used to acquire knowledge from the data and allow for terms that are not currently within an existing set of rules. Both a rules-based and an automatic system can also be combined to utilize an initial database as a reference while also allowing for the inclusion of new terms and the alteration of sentiment values [28].

### Step 1: Developing and Testing Our SA Algorithm

We developed a potential SA algorithm from an NLP library known as TextBlob. This library was built out of a toolkit using many different resources that are versatile and contain millions of training texts ranging from movie reviews to online conversations. TextBlob uses a naïve Bayes classifier which is a natural language toolkit (NLTK) that was trained from a movie review corpus. Millions of reviews were striped into tokens that were assigned positive or negative values to allow for the sentiment of the entire message to be interpreted.

Since naïve Bayes is a generative model while other approaches such as linear regression (LR) are discriminative, we felt that Naïve Bayes was a stronger model to use for a small data set which requires extending beyond the corpus that was originally used for training. This is only true if the assumption of independence holds, which is the case with our data. In addition, naïve Bayes performs well in the presence of categorical input variables, which is also the case in this study. Lastly, TextBlob is well documented and therefore is easy to integrate into our existing algorithm [29].

To determine the accuracy of our newly developed SA algorithm for our purposes, we utilized a pre-existing and de-identified data set of interviews with health professionals about their implicit biases. Ethics approval was not required for secondary analysis of de-identified data. We conducted SA on the transcribed interviews to score their underlying sentiment. We then compared the machine score with a manual human-scored sentiment categorization which had been completed prior to the algorithm execution. This comparison allowed us to determine the accuracy of the algorithm within the context of health professions' education and practice. We calculated the accuracy of our algorithm by calculating how many interviews were correctly computed in comparison to the manually scored value.

### Step 2: Application of SA to Twitter Archive

We collected an archive of publicly available tweets, including metadata such as display name, username, and user biography through the Twitter Application Programming Interface (API). These “tweets” were stored if they included specific hashtags, which are commonly used to discuss bias-related topics. The hashtags included were “#AllLivesMatter/#ALM,” “#BlackLivesMatter/#BLM,” “#HeForShe,” “#ImplicitBias,” “#RepresentationMatters,” and “#UnconsciousBias.”

Our archive was then categorized into two databases, “health professionals” and “general population .”We distinguished between each group by searching for specific markers in the display name, username, or biography that were manually checked to ensure all individuals included in the data set would fit the classification of health professionals. The individuals whose “tweets” belonged to the general population had no additional criteria to be met other than using the hashtag.

The data collection process was initiated with the first official data pull on 12 January 2020 and collected for approximately three months, commencing on 29 March 2020, when the database was sufficient enough to analyze. The final archive contained 555 “tweets” from health professionals and 6680 tweets from the general population.

To compare sentiment scores between health professionals and the general population, the total sums in each of the three categories, “positive,” “negative,” and “neutral” were calculated for each of the two databases, “health professionals” and “general population.” The purpose of the general population proportions was to serve as an expected value and to identify if health care professionals vary from this standard. This then allowed us to perform a chi-square goodness of fit test. We selected the chi-square goodness of fit test after methodological consultation with local experts in epidemiology and biostatistics. In general, a chi-square allows researchers to draw inferences and test for relationships between categorical variables. The goodness of fit test is useful to evaluate whether a full population is represented through the sample data. As our research sample sought to compare the sentiment between health professional discourse and the general population, we felt the goodness of fit test would be appropriate.

We noted that the volume of data being collected between the general population and health professionals was vastly different in quantity. We chose to use proportions as the quantity of data may have been misleading. As there were fewer health professional tweets included, we scaled down this group to have more tangible numbers for our statistical analysis. For example, on a given data pull, if there were 150/500 negative tweets from the general population versus 12/20 negative tweets from health professionals, the comparison of raw quantities would have skewed analysis and interpretation. Therefore, the observed values were comprised of the counts of each category in the health professional data set. The expected values were the proportion of each category in the general population data set scaled to the sum of the health professional data set. The standard significance value of .05 was maintained, and considering there were three categories, two degrees of freedom were present, and we concluded that an *χ*^2^ value of 9.21 was required for the deviation from the general population to be deemed statistically significant.

### Programming Specifications

Our SA algorithm was written in Python 3.0. This was an object-oriented program that used a class method to handle Twitter API credentials, authorize access to the database, and utilize the NLP as the “tweets” were retrieved. A class method refers to the structure of the algorithm, which means that the class, program code template, and method are bound to the class and not the object of the class. In programming, class refers to a descriptor of certain objects rather than the objects themselves. Our algorithm was developed into a Python script for each hashtag, and then a bash script file was written to allow ease of access to collect the data. A bash file refers to a text file that contains a series of commands. In this study, the bash file contained the commands to run the Python algorithms to collect data and populate the database. Overall, we used the same algorithm for both components of this study, accuracy testing and Twitter analysis. However, there were slight modifications, such as removing authentication from the local accuracy testing script as the data was retrieved locally.

## Results

In order to test the accuracy of the NLP library for the interviews conducted, there were 53 health professionals, including registered nurses and medical doctors. When we tested the original algorithm, our tool was able to accurately identify more than the required number of underlying sentiments to be deemed valid.

With 44 out of the 53 interviews (83%) being correctly assessed on sentiment with the utilization of the equation referenced, this returned an accuracy of 0.82, which was higher than the required threshold of 0.75. This concluded that using the TextBlob library was highly accurate but not subject to minor deviance. Nonetheless, it can still be utilized with high confidence when applied to a topic such as health care. Table 1 provides a breakdown of the scores.

When applying the algorithm to the tweets gathered, there was a noticeable difference in the sentiments between health care professionals and the general population. This discrepancy highlighted a smaller proportion of neutral tweets from health care professionals’ professional accounts on social media. This difference was proven to be statistically significant.

When using the chi-square test equation for goodness of fit, a *χ*^2^ value of 35.455 is achieved (*χ*^2^ [2, n= 555]=35.455; *P*<.001). As this value is higher than the 9.21 required for significance to be achieved, the results can be deemed statistically significant. Thus, it can be stated that health care professionals attach more sentiment to their posts on Twitter than seen in the general population. Table 2 provides a more detailed breakdown of the scores and comparison. Table 3 and Table 4 provide an illustration of the sentiment scores. Table 3 shows that the sentiment of health care professionals was more positive, less neutral, and less negative than expected. Table 4 shows the variance in the sentiment between the tweets between health care professionals and the general population of tweets with the same specified hashtags. This figure suggests that tweets by health professionals were more positive, less negative, and approximately the same level of neutrality when compared to the general population of tweets. Table 5 provides a breakdown of sample tweets.

**Table 1:** The sentiment algorithm, TextBlob, on interviews of health care professionals regarding implicit bias in medicine

Group	Total interviews	Correctly scored	Accuracy
Pediatric physician	11	10	0.90
Pediatric nurse	10	8	0.80
Psychiatric nurse	11	10	0.90
Psychiatric resident	10	7	0.70
Psychiatric physician	11	9	0.82
Total	53	44	0.83

**Table 2:** The sentiment score of the tweets by health professionals and their associated chi-square values with intermediaries.

Sentiment Score	Observed	Expected	Difference	Difference Sq.	Difference Sq./Exp Fr.^a^
Positive	275.00	211.18	63.82	4073.3773	19.2889
Negative	42.00	70.70	–28.70	823.5057	11.6484
Neutral	238.00	273.13	–35.13	1233.8518	4.5175
**Total**					35.455^b^

^a^Expected Fraction.

^b^Chi-square statistic value which is used to determine statistical significance.

**Table 3:** The observed and expected values for sentiment score of the tweets by health professionals.

	Observed	Expected
Positive	275	211.18
Negative	42	70.7
Neutral	238	273.13

**Table 4:** The variance in sentiment between health care professionals and the general population with respect to the same hashtags.

	Health Professionals	General Population
Positive	45.66	38.22
Negative	5.2	12.74
Neutral	49.14	4904

**Table 5:** Sample tweets from health care professionals and the general population with equity-related hashtags.

	Positive	Neutral	Negative
Health professionals	*It’s #WomensHistoryMonth and #AMWA will be spotlighting incredible #womeninmedicine all month-long!*	*Please join us at the diversity in medicine conference #docswithdisabilities*	*Maybe if I work hard enough and almost die of COIVD my patients will start calling me “doctor” instead of “mademoiselle” #genderbias #womeninmedicine*
General population	*Moving from a safe place to a brave place to address issues of #implicitbias #CCM49 Great session thank you!*	*Check out this in-depth podcast…on educating scientific communities #implicitbias*	*Gender bias is not a good look #genderbias #checkyourgenderbias #unconsciousbias*

## Discussion

### Principal Findings

The finding that health professionals are more likely to show and convey emotions regarding equity-related issues on social media has implications for teaching and learning about sensitive topics related to equity and bias for health professionals. Such emotions are likely to influence learning processes and therefore must be considered in the design, delivery, and evaluation of equity and bias-related education.

### Emotions and Identity in Health Professions Education

Our aims through this research were to gain further insight into how emotions influence equity and bias-related education through SA. By leveraging advances in ML technology, NLP, and SA, we developed, tested, and applied a novel SA algorithm to social media discourse. Our findings suggest that health professionals are more likely to convey emotions on social media about equity-related topics than the general public. Although previous research has found evidence that there are defensive reactions to discussions about bias among both health professionals and the general public [30-32], our SA findings suggest that health professionals may be uniquely susceptible to defensiveness and counter-react through positive emotion as a response.

This finding aligns with previous research on defense mechanisms to grapple with the reality of an individual’s role in perpetuating prejudice or discrimination [33]. Our study suggests the evidence of reaction formation as a defense for learners. Reaction formation refers to when an individual forms an attitude that is the opposite of one’s threatening or unacceptable actual thoughts [34]. By conveying a higher degree of positive sentiment, health professionals may be attempting to project that they are more neutral or objective when, in reality, they demonstrate the same degree of bias as the general population [35].

We also found that variance in sentiment between health professionals and the public suggests that not only do health professionals convey more emotion, but they also demonstrate greater sentiment variance related to positive emotion compared to the general public, who convey greater variance related to negative emotion. Greater positive sentiment among health professionals suggests that health professionals are utilizing Twitter differently than the general public. Therefore, our findings suggest caution for health professions educators who attempt to challenge normative thinking of health professionals as neutral or objective. Skilled facilitators may be necessary to mediate and regulate emotions among both teachers and learners when such challenges arise [36].

### Emotions and Social Media

Social media discourse provides an opportunity to explore how individuals react to social issues and world events. Tweets provide a source of data that can be automatically classified according to sentiment to provide insights into the emotional nature of certain topics. Although SA has been previously used for digital marketing or opinion mining, its use in health professions education research has been to date quite limited.

Global events and social movements related to equity and bias, such as #BlackLivesMatter and #JusticeforGeorgeFloyd, underscore the importance of social media discourse as it relates to teaching and learning about bias. Such reactions among both health professionals and the general public during unexpected events can provide evidence for collective sense-making [37], social sharing of emotions [38], and individual strategies of approach/avoidance [39]. Emotions also mediate how contact between and among different social groups can effectively address prejudice [40]. As we set out to explore in our study, SA may be an effective tool to analyze such discourse.

Before it can be effectively applied, however, the limitations of SA require ensuring its accuracy and utility in a health professions education context. Our study provides an example of a SA algorithm that was tested for accuracy before being applied. This algorithm can be used in future research to analyze sentiment associated with social media discourse and may also have future applications to other types of archives such as electronic health records.

### Sentiment Analysis in Health Professions Education

Advances in NLP applied to textual data for educational purposes are developing at a rapid pace. SA has demonstrated potential in evaluating instruction, designing policy, enhancing learning systems, and educational research [41]. For example, SA has been used to analyze students’ feedback to improve teaching [42-44] and track students’ emotions across longitudinal learning activities through learning diaries [44]. However, there is a paucity of research into how SA can be applied specifically in a health profession education context.

Our study provides an example and template for future researchers to develop and utilize SA for a variety of purposes. We also hope that our work can provide insights into the emotionally charged nature of teaching and learning about bias and inform future work to develop, implement, and evaluate antibias and antiracism curricula for health professions learners.

### Key Implications and Future Directions

For health professions educators to effectively consider emotions in the design, delivery, and evaluation of equity or bias-related curricula, educators should anticipate defensive reactions when emotions are provoked and ensure skilled facilitation for sensitive or emotionally charged discussions. Our finding regarding the unique nature of social media discourse among health professionals and the public also suggests that health advocacy curricula must be augmented with information on digital aspects of advocacy. In addition, existing teaching and learning on digital professionalism may benefit from information regarding sentimentality and how digital aspects of communications differ from traditional media.

### Limitations

A key limitation of a SA approach is that SA focuses on categorical aspects of sentiment value such as positive, negative, or neutral. This limits our ability to understand nuanced emotional states that reflect an individual’s experience. Past research on how individuals cope with potentially threatening feedback related to their biases highlights that ambivalence may form an important component of how they can respond to identity threats and move forward towards change [45]. Additional research is therefore needed, particularly into how situations are perceived and the individual and social resources that individuals experience or have to cope with emotions that may interfere with learning.

Another important limitation of using NLP is that it requires the classification model to be trained. This requires intensive learning and manual categorization, and the most accurate models are still continuing to improve. However, the most efficient models have not been trained to categorize health care–specific data. While this research has proven a high accuracy rate (0.83), it must be recognized it is not all-encompassing and open to errors. Nonetheless, accuracy will only continue to improve, and in turn, these models will become more relevant. It will be important to ensure that health care data is used in these training processes.

Our study was conducted in 2019 when BERT (Bidirectional Encoder Representations from Transformers) models were less commonly used. Although such models allow for better sentence processing leveraging the architecture for the Corpus of Linguistic Acceptability, they require an extremely large corpus of testing data for models, which would not necessarily align with our criteria and potential limit accuracy.

Further, it is worth noting that an unavoidable bias exists within any NLP algorithm itself as a human-designed approach may be subject to the biases present in the training data set. This is an area of future work that should be considered as SA in health education evolves with larger data sets.

Lastly, we recognize that our SA was not developed using data from the general public; however, we believed that it would be reasonable to use on general public tweets due to previous research on defensive reactions to bias-related feedback in the general public that align with our previous studies, and other research.

### Conclusions

To explore the role of emotions in teaching and learning about bias and equity for health professionals, we developed and tested a SA algorithm of bias-related discourse. We developed a highly accurate SA algorithm that demonstrated health professionals use a higher degree of emotion when communicating about bias on social media compared to the general population. Our findings support that emotions must be considered in the design, delivery, and evaluation of equity and bias-related education.

## Abbreviations

API: application programming interface
ML: machine learning
NPL: natural language processing
SA: sentiment analysis
